# Finding Static Stability Limits: Comparison of Reactive Balance in Older People With and Without a History of Falls

**DOI:** 10.1093/geroni/igab046.1726

**Published:** 2021-12-17

**Authors:** Michel Hackbarth, Jessica Koschate, Sandra Lau, Tania Zieschang

**Affiliations:** Carl von Ossietzky University Oldenburg, Oldenburg, Niedersachsen, Germany

## Abstract

Reactive balance is a highly relevant fall risk factor, but is rarely considered in clinical practice. Especially medio-lateral perturbations lead to a pronounced instability of the gait pattern. However, there is no consensus on a method for the assessment of individually challenging perturbation intensities to apply during walking. The aim of this study is to determine and compare the static stability-limits in older adults with and without a history of falls. Twelve older adults with (OAF; 75.6 ±3.66,9♀) and 19 older adults without a history of falls (OA; 77.5 ±4.99,12♀) were subjected to progressive-intensifying perturbations while standing on a perturbation treadmill. In addition, functional performance (Mini-BESTest), fear of falling (FES-I), and physical activity (kcal) were assessed Deflection of the treadmill-platform was randomized by timing and direction and was increased until the subject had to compensate with a step (stability-limit). The maximum deflection distance for each direction, as well as the FES-I score, mini-BESTest score, and activity level were evaluated for group differences using the t-test and Mann-Whitney-U test (α≤5%). There were no significant group differences in the mini-BESTest and between the maximum tolerated deflection distances. The OAF-subjects showed an increased FES-I score (median for OA=18.0 and OAF=22.0, p=0.032) and higher activity levels (median for OA=1974 kcal and OAF=3365 kcal, p=0.011). Despite an increased fear of falling, the older adults with a fall history showed a similar stability-limit, but higher activity levels. In future experiments these static stability limits should be tested during walking and evaluated via motion analysis.

